# N Addition Overwhelmed the Effects of P Addition on the Soil C, N, and P Cycling Genes in Alpine Meadow of the Qinghai-Tibetan Plateau

**DOI:** 10.3389/fpls.2022.860590

**Published:** 2022-04-26

**Authors:** Jiannan Xiao, Shikui Dong, Hao Shen, Shuai Li, Kelly Wessell, Shiliang Liu, Wei Li, Yangliu Zhi, Zhiyuan Mu, Hongbo Li

**Affiliations:** ^1^School of Environment, Beijing Normal University, Beijing, China; ^2^School of Grassland Sciences, Beijing Forestry University, Beijing, China; ^3^College of Resource and Environment, Shanxi Agricultural University, Taigu, China; ^4^Tompkin Cortland Community College, Ithaca, NY, United States; ^5^Institute of Environment and Sustainable Development in Agriculture, Chinese Academy of Agricultural Sciences, Beijing, China

**Keywords:** microbial functional gene, nitrogen addition, phosphorus addition, biogeochemical cycling, alpine grassland

## Abstract

Although human activities have greatly increased nitrogen (N) and phosphorus (P) inputs to the alpine grassland ecosystems, how soil microbial functional genes involved in nutrient cycling respond to N and P input remains unknown. Based on a fertilization experiment established in an alpine meadow of the Qinghai-Tibetan Plateau, we investigated the response of the abundance of soil carbon (C), N, and P cycling genes to N and P addition and evaluated soil and plant factors related to the observed effects. Our results indicated that the abundance of C, N, and P cycling genes were hardly affected by N addition, while P addition significantly increased most of them, suggesting that the availability of P plays a more important role for soil microorganisms than N in this alpine meadow ecosystem. Meanwhile, when N and P were added together, the abundance of C, N, and P cycling genes did not change significantly, indicating that the promoting effects of P addition on microbial functional genes abundances were overwhelmed by N addition. The Mantel analysis and the variation partitioning analysis revealed the major role of shoot P concentration in regulating the abundance of C, N, and P cycling genes. These results suggest that soil P availability and plant traits are key in governing C, N, and P cycling genes at the functional gene level in the alpine grassland ecosystem.

## Introduction

Nitrogen (N) is a common nutrient element limiting plant productivity in most terrestrial ecosystems (Vitousek and Howarth, 1991). Atmospheric N deposition has increased substantially over the last decades due to anthropogenic activities (Liu et al., 2015) and has dramatically altered the characteristics of soils and plants (Pan et al., 2014). For example, studies have shown soil acidification and eutrophication, reduced species diversity, and increased primary productivity under N deposition (Jing et al., 2016; She et al., 2018; Li et al., 2019). Phosphorus (P) is another key nutrient for plant growth and ecosystem health, but P has not increased by anthropogenic activity to the same degree as N (Penuelas et al., 2013). N deposition may result in the limitation of P by influencing the nutrient balance in the soil (Cui et al., 2020).

Soil microorganisms play a major role in driving carbon (C), N, and P biogeochemical cycles and in responses to environmental changes (Lynn et al., 2016; Smercina et al., 2021). Functional genes, encoding various enzymes, not only provide important information about the genetic potentials for specific metabolic processes in soil but also reflect the diversity and activity of microbial communities (He et al., 2012; Turner et al., 2019). Nutrient availability is a key player in shaping soil microbial community (Lauber et al., 2008). N and P addition that increase N and P availability are responsible for changing the abundance and the community structure of soil microorganisms (Wang et al., 2017). The response of soil microbes to N addition has been widely reported across ecosystems. Generally, N addition tends to decrease the abundance of microbial community due to reduced belowground C allocation under N addition (Frey et al., 2004; Bae et al., 2015). N addition decreased the gene abundance of ammoxidation (*AOA*) and N fixation (*nifH*) (He et al., 2010) and increased the abundance of *narG*, *nirK*, *nirS*, and *nosZ* genes (denitrification) (Kastl et al., 2015). In a temperate steppe ecosystem, 5-year of N addition enhanced the abundance of the *AOB* gene by 11-fold and decreased the abundance of *nifH*, *chiA*, *AOA*, *nirS*, *nirK*, and *nosZ* genes by 30–50% (Zhang et al., 2019). N addition (exceeding 40 kg N ha^–1^ year^–1^) notably enhanced the abundance of C degradation genes in an alpine steppe (Chen et al., 2020). Similar to N addition, P addition can alter the structure of microbial functional genes (Tan et al., 2013). Both increased and decreased soil microbial diversity under P fertilization have been reported in various ecosystems (Chhabra et al., 2013; Tan et al., 2013). These inconsistent results may be due to the fact that P input may not be the only factor responsible for changes in the microbial community and that other environmental factors such as soil characteristics and climate may also be involved in the process (Tan et al., 2013).

It is noteworthy that N and P additions not only put a direct influence on microbial community but also indirectly affected microbial abundances through vegetation (Li et al., 2017; She et al., 2018). Plant traits can alter soil abiotic factors that regulate the abundance, composition, and activity of soil microbial communities (Abalos et al., 2019). N and P additions can influence plant growth, leading to the alteration of plant residue, such as litter and root exudate, which directly affect the structure and the composition of the microbial community (She et al., 2018). For example, with the increase of belowground plant biomass after P addition, the plant C distribution to soil was enhanced, hence improving microbial biomass and microbe phenotype (Dong J. et al., 2020). Aboveground plant biomass indirectly stimulated the abundance of soil microbial functional genes by increasing soil dissolved organic carbon (Li et al., 2017). To date, the impacts of N and P additions, as well as their complex interactive influences on the microbial mediation of C, N, and P biogeochemical cycling in alpine grassland ecosystems, remain elusive, despite the fact that alpine grassland ecosystems are globally vital in providing ecosystems services and functions such as C/N storage, biodiversity conservation, water conservation, and nutrient cycling (Dong et al., 2007).

The Qinghai-Tibetan Plateau (QTP) is the largest plateau in China and the highest in the world, and it is described as a “Global Change Warning Area” and the “Global Climate Stove” due to its sensitivity to global change (Dong et al., 2007). This region has been experiencing a significant increase in N deposition, approximately 8 kg N ha^–1^ year^–1^ (Lü and Tian, 2007). Due to its low contents and decomposition rates, N is always considered to be the critical limiting factor of alpine ecosystems on the QTP (Li et al., 2019). However, it has also been reported that P, not N, is the limiting factor in the QTP’s alpine grassland ecosystem (Jing et al., 2016). Therefore, it has not been determined whether the alpine ecosystems on the QTP are limited by either N, P, or N and P. N and P inputs could affect ecosystem function significantly (Jing et al., 2016). Elevated N deposition has significantly influenced soil properties and vegetation characteristics of QTP’s alpine grasslands (Jiang et al., 2013; Ma et al., 2016; Li et al., 2019; Chen et al., 2020). P addition suppressed topsoil microbial activity and increased plant primary productivity in an alpine grassland (Jing et al., 2016). Alpine meadow, which is widely distributed on the QTP, accounts for over 40% of the QTP’s territory (Kang et al., 2007) and provides essential ecosystem functional services for millions of people living there and downstream (Dong et al., 2007). However, to our knowledge, few researchers have documented the response of soil C, N, and P cycling genes to N and P addition in QTP’s alpine meadow, and the environmental factors that shape these genes in soil have not yet been established. These uncertainties will restrict our ability to predict the response of the biogeochemistry cycle to future environmental changes in the QTP’s alpine ecosystems.

Herein, we measured the abundances of soil microbial functional genes for C fixation (*smtA*, *rbcL, mct*, *pccA*, *acsE*, *acsA*, *aclB*, *frdA*, *korA*, and *accA*), C degradation (*amyA*, *apu*, *manA*, *abfA*, *CDH*, *chiA*, *exg*, *gam*, *glx*, *IsoP*, *lig*, *mnp*, *exoPG*, and *xylA*), N cycling (*amoA*, *amoB*, *hzsB*, *nirK*, *nirS*, *nosZ*, *nxrA*, *nifH*, *napA*, *gdh*, and *UreC*), and P cycling (*phnK*, *pqqC*, *bpp*, *phoD*, *phoX*, *ppx*, *gmGDH*, and *emGDH*). We also measured plant biomass and nutrients and soil nutrients to analyze their relationships with C, N, and P cycling genes under N and P additions. We hypothesized that (1) combined N and P additions would promote the functional capabilities of C, N, and P cycling genes in this alpine meadow ecosystem by elevating nutrient availabilities; (2) its promotion effect would be greater than that of N alone addition due to the alleviation of P limitation; and (3) N and P additions not only put direct influence on microbial functional genes but also indirectly affect them through vegetation.

## Materials and Methods

### Site Description and Experimental Design

**FIGURE 1:**
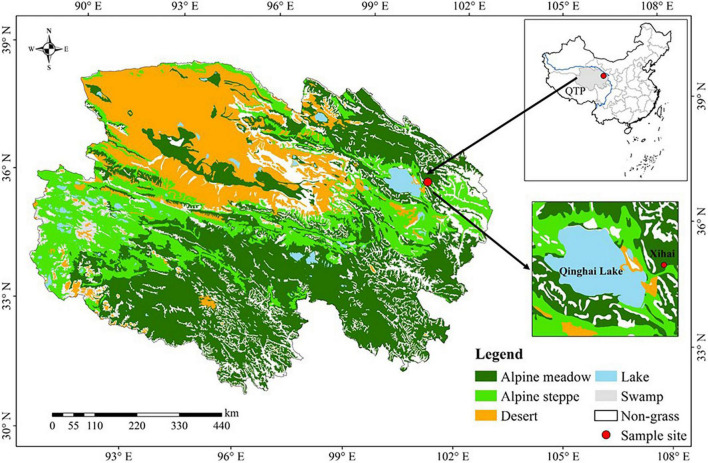
The location of study site.

This study was carried out in Xihai Town of Haiyan County (36°56′N, 100°57′E, 3100 m ASL) of the Qinghai Province, China (Figure 1). The major vegetation in Haiyan County was classified as alpine meadow, and the dominant plant species include *Agropyron cristatum*, *Poa pratensis*, *Elymus dahuricus*, and *Stipa capillata* (Li et al., 2019). The mean annual temperature and mean annual precipitation at Haiyan County were 1.4°C and 330–370 mm, respectively. The mean daily temperature and precipitation during the growing season are shown in Supplementary Figure 1. The soil was classified as Mat-Gryic Cambisols (Chinese National Soil Survey), with sand, silt, and clay contents of 16.75, 58.7.3, and 24.55%, respectively.

In May of 2019, the field experiment was established using a randomized block design (2 m × 5 m plots separated by a buffer of 1 m). In brief, 12 plots were assigned to four treatments with 3 replications. The four treatments were CK (no fertilizer), N (72 kg N hm^–2^), P (36 kg P hm^–2^), and NP (72 kg N hm^–2^ + 36 kg P hm^–2^). The N addition level (72 kg N ha^–1^ year^–1^) sufficiently simulated N critical loads (50 kg N ha^–1^ year^–1^ reported by Zong et al., 2016). N added as ammonium nitrate (NH_4_NO_3_) and P added as calcium superphosphate [Ca(H_2_PO_4_)_2_] were applied in May, July, and September each year since 2019. The same amount of N and P fertilizers were applied each time. NH_4_NO_3_ and Ca(H_2_PO_4_)_2_ were weighed, mixed with 0.5 kg sand, and evenly distributed to each plot by hand after sunset (for higher moisture).

### Plant and Soil Sampling and Analyses

In mid-August 2020, aboveground biomass (AGB) and belowground biomass (BGB) were measured at the peak of plant growth. We clipped all aboveground plants at the ground level from a 0.25 m × 0.25 m quadrat in each plot and took 3 soil cores (3.5 cm diameter × 20 cm depth) in the clipped area. The soil cores were soaked in water to separate the roots using a 0.5 mm mesh. The collected plant samples were oven-dried at 65°C to constant weight and weighed as AGB and BGB.

Soil samples were collected in the clipped area using a 3.5 cm diameter corer at depths of 0–20 cm in mid-August 2020. Fresh soil was immediately processed to determine soil moisture, and the remaining soils of each sample were divided into three parts. One part was air-dried for the determination of total nutrients, another part was stored at 4°C for the determination of available nutrients, and the remaining part was stored at −80°C for DNA extraction.

An element analyzer (EA 3000, Italy) was used to measure total C and N concentration in plant shoot (C_shoot_ and N_shoot_), root (C_root_ and N_root_), and soil (TC and TN), and an ICP-AES analyzer (Thermo-Jarrell Ash Corp., MA, United States) was used to measure total P concentration in plant shoot (P_shoot_), root (P_root_), and soil (TP). A flow injection auto-analyzer (AACE, Germany) was used to measure soil NH_4_^+^ and NO_3_^–^, and inductively coupled plasma spectrometers (ICP; SPECTRO ARCOS EOP, Germany) were used to measure the soil’s available phosphorus (AP).

### Microbial DNA Extraction and Quantitative PCR Analysis

Soil DNA was extracted from 0.5 g soil using a FastDNA Spin kit for soil (MP Biomedicals, United States) by following the manufacturer’s instructions. DNA quality was assessed using a spectrophotometer (NanoDrop ND-1000, Thermo Scientific, Waltham, MA, United States). DNA concentrations were quantified with a QuantiFluor dsDNA system (Promega, Madison, WI, United States) using a microplate reader (Spectramax M5, United States). DNA was stored at −20°C for later use.

Furthermore, 48 primer sets targeting genes were used to characterize the soil DNA, including 24 genes involved in C cycling, 16 genes in N cycling, 8 genes in P cycling, and 1 reference gene (16S rRNA) (Supplementary Table 1). High-throughput qPCR was used in this study to detect the copy numbers of these genes. Triplicate amplicons were analyzed using the Wafergen SmartChip Real-time PCR system (Wafergen, Fremont, CA, United States) as follows: 95°C for 10 min, followed by 40 cycles of 95°C for 30 s, 60°C for 30 s, and 72°C for 30 s. The melting curve was automatically generated by WaferGen software. Results with multiple melting peaks or amplification efficiencies < 90% and > 110% were discarded by the SmartChip qPCR software. A threshold cycle (CT) < 31 was used for subsequent analysis. The gene absolute copy number was calculated according to Eq. 1, and the gene relative copy number was calculated according to Eq. 2, as described by Dong S. et al. (2020).


(1) $$\mathrm{Gene}\,\mathrm{absolute}\,\mathrm{copy}\,\mathrm{number}=10^{\left(31-{\text{C}}_{\text{T}}\right)/\left(10/3\right)}$$

(2) $$\mathrm{Relative}\,\mathrm{copy}\,\mathrm{number}=\frac{{\text{CN}}_{\text{i}}}{{\text{CN}}_{16\,S}},$$

where C_T_ is the threshold cycle, CN_i_ is the copy number of one of the 48 genes, and CN_16S_ is the copy number of the 16S rRNA gene.

### Statistical Analysis

A one-way ANOVA was performed to identify significant differences in microbial functional genes, soil nutrients, and plant biomass and nutrients in response to N and P addition. A two-way ANOVA was performed to test the effects of N addition, P addition, and their interactions on soil C, N, and P cycling genes. Principal coordinates analysis (PCoA) based on the Bray–Curtis distances was applied to evaluate the differences of functional genes among experimental treatments. Non-parametric multivariate statistical analyses, including multi-response permutation procedures (MRPP), analysis of similarity (ANOSIM), and permutational multivariate analysis of variance (ADONIS) were used to determine the dissimilarity of microbial functional gene abundance among different treatments. Pearson correlations were performed to analyze the relationships between microbial functional genes and plant and soil nutrients. The Mantel test was conducted to examine the correlations between microbial functional genes and soil and plant variables. The variation partitioning analysis (VPA) was performed to determine the relationships between soil and plant variables and microbial functional genes. All statistical analyses were performed using the R Development Core Team. “vegan” and “corrplot” package (version 4.0.4).

## Results

### Impacts of N and P Addition on Microbial Functional Genes

**FIGURE 2:**
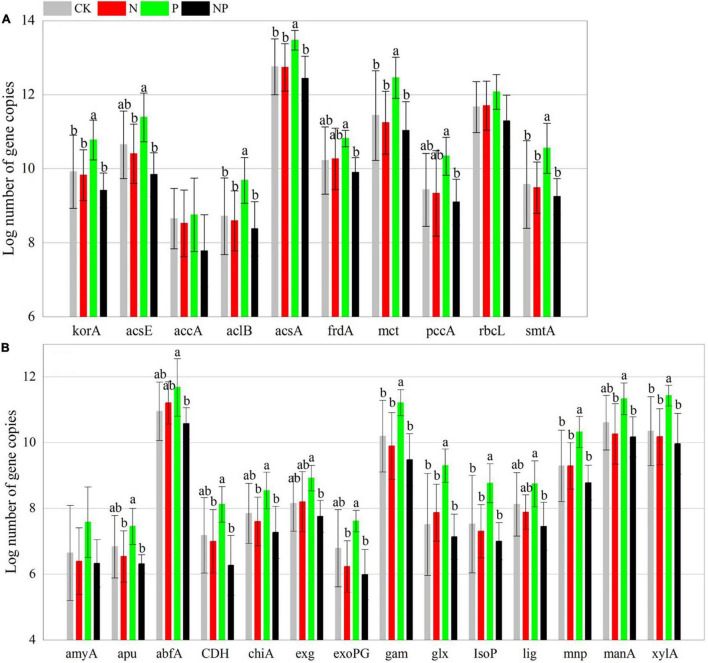
Abundance of C fixation **(A)** and C degradation **(B)** genes among experimental treatments. Different letters indicate significant differences at a *p* < 0.05 level across experimental treatments. No letter indicates a non-significant difference (CK = control, N = addition of 72 kg N hm^–2^ year^–1^, P = addition of 36 kg P hm^–2^ year^–1^, NP = interaction of N and P).

**FIGURE 3:**
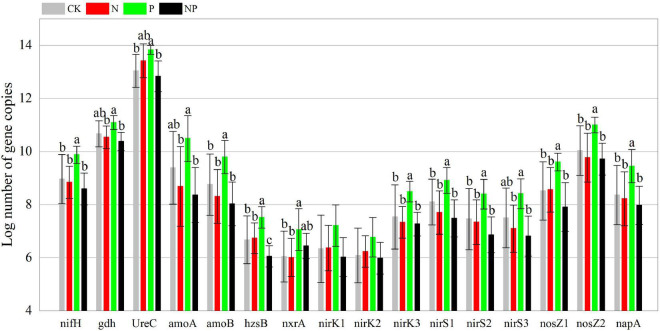
Abundance of N cycling functional genes among experimental treatments. Different letters indicate significant differences at a *p* < 0.05 level across experimental treatments. No letter indicates a non-significant difference (CK = control, N = addition of 72 kg N hm^–2^ year^–1^, P = addition of 36 kg P hm^–2^ year^–1^, NP = interaction of N and P).

**FIGURE 4:**
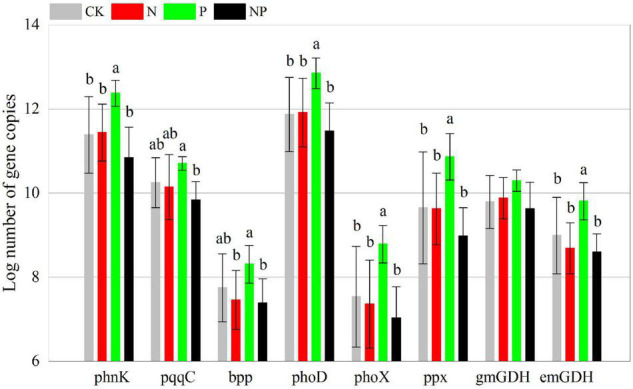
Abundance of P cycling functional genes among experimental treatments. Different letters indicate significant differences at a *p* < 0.05 level across experimental treatments. No letter indicates a non-significant difference (CK = control, N = addition of 72 kg N hm^–2^ year^–1^, P = addition of 36 kg P hm^–2^ year^–1^, NP = interaction of N and P).

Phosphorus addition had significant effects on 39 of the 48 C, N, and P cycling genes (Supplementary Table 2). In contrast to CK, P alone addition significantly (*p* < 0.05) increased the abundance of 24 functional genes (*korA*, *aclB*, *acsA*, *mct*, *smtA*, *gam*, *glx*, *IsoP*, *mnp*, *xylA*, *nifH*, *amoB*, *hzsB*, *niK3*, *nirS1*, *nirS2*, *nosZ1*, *nosZ2*, *napA*, *phnK*, *phoD*, *phoX*, *ppx*, and *emGDH*) but had no significant effects (*p* > 0.05) on the other 24 functional genes. N and NP additions had insignificant (*p* > 0.05) effects on all functional genes (Figures 2–4). However, there were interactive effects between N and P on 35 of the 48 C, N, and P cycling genes (Supplementary Table 2).

**FIGURE 5:**
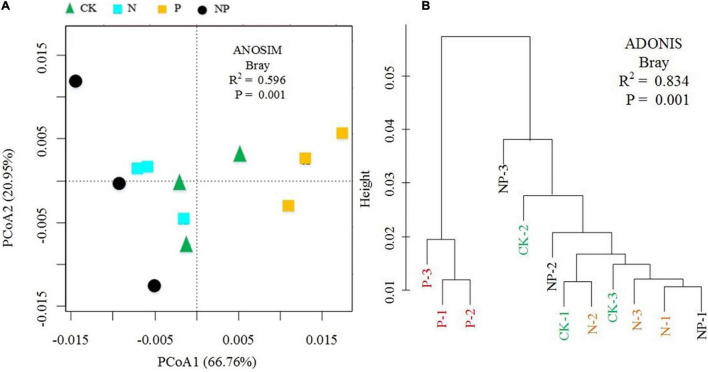
**(A)** Principal coordinates analysis (PCoA) and **(B)** cluster analysis of microbial functional gene composition (CK = control, N = addition of 72 kg N hm^–2^ year^–1^, P = addition of 36 kg P hm^–2^ year^–1^, NP = interaction of N and P).

The PCoA plot revealed a distinct separation in β diversity by N and P addition, with the first two axes explaining 66.76% (axis 1) and 20.95% (axis 2) (Figure 5A). The cluster analysis showed that C, N, and P cycling genes of P addition were clearly separated from those under other treatments (CK, N, and NP) (Figure 5B). Non-parametric multivariate dissimilarity tests (MRPP, Adonis, and ANOSIM) further indicated that the differences in the pattern of C, N, and P cycling genes were significant (Supplementary Table 3).

### Impacts of N and P Additions on Soil and Plant Variables

**FIGURE 6:**
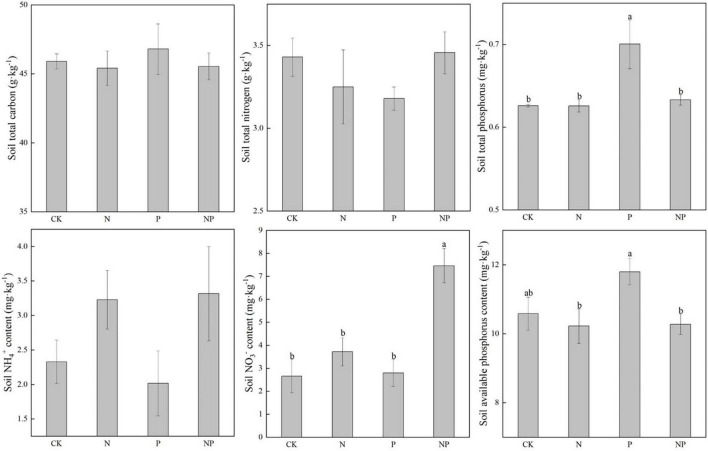
Soil nutrients under different experimental treatments. Different letters indicate significant differences at a *p* < 0.05 level across experimental treatments. No letter indicates a non-significant difference (CK = control, N = addition of 72 kg N hm^–2^ year^–1^, P = addition of 36 kg P hm^–2^ year^–1^, NP = interaction of N and P).

Compared with CK, N, and P additions had no significant effects on soil TC, and TN, NH_4_^+^, and AP. Soil total P (TP) and NO_3_^–^ increased significantly under P addition (*p* < 0.05) (Figure 6).

**FIGURE 7:**
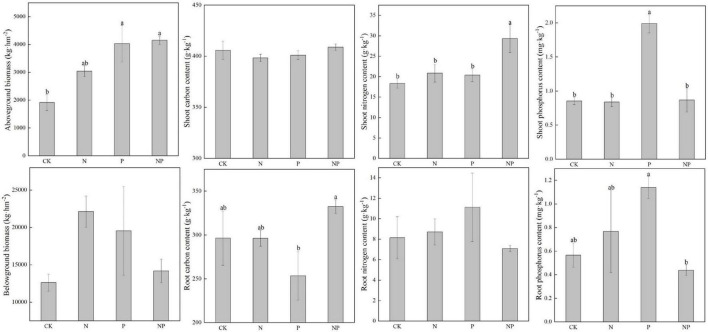
Plant biomass and nutrients under different experimental treatments. Different letters indicate significant differences at a *p* < 0.05 level across experimental treatments. No letter indicates a non-significant difference (CK = control, N = addition of 72 kg N hm^–2^ year^–1^, P = addition of 36 kg P hm^–2^ year^–1^, NP = interaction of N and P).

N alone addition showed no significant effects on plant biomass and nutrients (*p* > 0.05, Figure 7). P alone addition significantly (*p* < 0.05) increased P_shoot_. Combined N and P addition significantly (*p* < 0.05) increased N_shoot_.

### Relationship Between Microbial Functional Genes and Environmental Variables

Most C, N, and P cycling genes were positively correlated with P_shoot_ and negatively correlated with C_root_ (*p* < 0.05, Supplementary Figures 2–4). A few functional genes were positively correlated with NH_4_^+^, AP, TP, and P_root_ and were negatively correlated with NO_3_^–^ and N_shoot_ (*p* < 0.05). N_root_, C_shoot_, TN, and TC were not significantly correlated with all microbial functional genes (*p* > 0.05). It showed that P, whether in plants or in soil, was positively correlated with microbial functional genes (Supplementary Figures 2–4).

The Mantel test showed that the functional genes were significantly correlated with NO_3_^–^, P_shoot_, and N_shoot_ (*p* < 0.05). Additionally, plant and soil variables were independently tested with the C, N, and P cycling genes. The C, N, and P cycling genes were all significantly (*p* < 0.05) correlated with NO_3_^–^ and P_shoot_, and the C cycling genes were also significantly correlated with N_shoot_ and C_root_.

**FIGURE 8:**
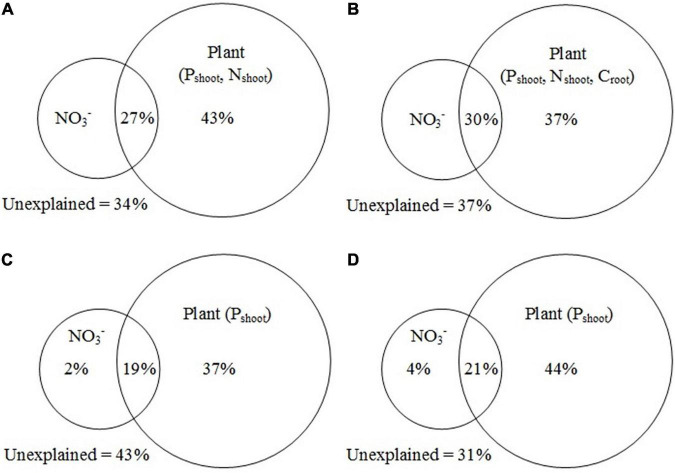
Variation partitioning analysis (VPA) of all functional genes **(A)**, C cycling genes **(B)**, N cycling genes **(C)**, and P cycling genes **(D)** explained by soil and plant variables. N_shoot_, N concentration of plant shoot; P_shoot_, P concentration of plant shoot; C_root_, C concentration of plant root.

The results from VPA showed that, for all functional genes, plant variables (P_shoot_ and N_shoot_) alone explained 43% of the variance, and the interaction between plant variables, and NO_3_^–^ explained 27% of the variance (Figure 8A). For C cycling genes, plant variables (P_shoot_, N_shoot_, and C_root_) alone accounted for 37% of the variance, while the interaction between plant variables and NO_3_^–^ accounted for 30% of the variance (Figure 8B). For N cycling genes, plant variables (P_shoot_) and NO_3_^–^ explained 37 and 2% of the variance, respectively, and the interaction between plant variables and NO_3_^–^ accounted for 19% of the variance (Figure 8C). For P cycling genes, plant variables (P_shoot_) and NO_3_^–^ contributed to 44 and 4% of the variance, respectively, and the interaction between plant variables and NO_3_^–^ contributed to 21% of the variance (Figure 8D). The Mantel test and the VPA results showed that P_shoot_ and NO_3_^–^ were the main factors influencing the C, N, and P cycling genes.

## Discussion

### Neutral Effects of N Addition on Microbial Functional Genes

All C, N, and P cycling genes showed a consistent neutral response to N addition, which did not support our first hypothesis and was inconsistent with previous scholars’ results (Zhang et al., 2019; Chen et al., 2020; Xiao et al., 2020). For example, N addition significantly increased the abundance of nitrifiers and denitrifiers in a meadow steppe (Xiao et al., 2020) of C degradation genes in an alpine steppe (Chen et al., 2020), whereas the neutral effects of N addition on the abundance of *nifH* and *nirS* have also been reported (Sun et al., 2015). Zhang et al. (2019) reported that N addition decreased the abundance of *nifH*, *chiA*, *AOA*, *nirS*, *nirK*, and *nosZ*. These discrepancies may be attributed to the differences in climate, soil properties, and vegetation characteristics in specific sites. In this study, we proposed two possible explanations for the results of this study.

One non-exclusive explanation refers to the N condition of this alpine meadow. The neutral effects of N addition on microbial functional genes in this study reiterate that N may be not limiting factors in this alpine ecosystem, corroborating previous findings reported from another alpine ecosystem (Jing et al., 2016). The background of N deposition (8 kg N ha^–1^ year^–1^) at this region over the past decades may have provided adequate N for the alpine plants and soil microbes (Lü and Tian, 2007). Once N saturation is reached, that is, exceeds the threshold of demand by plants and soil microbes, the additional N input no longer increases soil microbial abundance and diversity (Zhang et al., 2017). The insignificant responses of plant biomass (AGB and BGB) (Figure 7) and soil microbial biomass (Xiao et al., 2022) to N addition in the present study further proved that this alpine meadow ecosystem may be not limited by N.

Another plausible explanation may be related to the short-term study durations. The short timescale (2 years) of this study may hinder our examination of significant changes in microbial functional genes. The timescale over which to detect the response of soil microbial community to nutrient input is important (Jing et al., 2016). Allison and Martiny (2008) have reported that an average of 8 years was required before the microbial community significantly responded to fertilization. Rinnan et al. (2007) also demonstrated that, in contrast to significant responses after 15 years of fertilization to a tundra heath, 5, 6, and 10 treatment years had slight effects on microbial community composition, indicating the need for at least 10 years for the appearance of significant responses. The present study began in May 2019 and therefore the results represent the first 2 years after N addition. Several more years might be needed before a notable shift in the microbial community after the given N addition, highlighting the necessity for a long-term experiment for the reliable prediction of N deposition effects.

### Positive Effect of P Addition on Soil Microbial Functional Genes

Consistent with our first hypothesis, P alone addition strikingly increased the abundance of most microbial functional genes, indicating the particular sensitivity of the soil microbial community to P input. However, inconsistent with our second hypothesis, we observed non-significant effects of the combined addition of N and P on the abundance of microbial functional genes. The PCoA and the cluster analysis also showed that P treatment clustered together and distinguished from CK, N, and NP treatment, further confirming the limitation of P to soil microbial community in this alpine ecosystem. Similar influence and limitation of P on soil microbial communities in other ecosystems have also been observed (Jing et al., 2016; Wang et al., 2018; Li et al., 2020; Zou et al., 2020). Wang et al. (2018) reported that P addition increased the abundance of soil *nifH* gene in a fir plantation. Li et al. (2020) observed significant correlations between the abundance of C, N, and P cycling genes and the different levels of P amendments.

In alpine ecosystems, soil P is considered a limiting nutrient for plant growth due to the low decomposition rate (Jiang et al., 2013). P can be critical to soil microorganisms when it is limited (Zou et al., 2020). Appropriate P input can alleviate plants’ P limitation, promote plant growth, and then enhance rhizosphere secretions and C input to the soil, and the increased soil C from plant subsequently provide more substrates for soil microbes (Li et al., 2017; Jing et al., 2019). In this study, we found that P addition enhanced most of the C, N, and P cycling genes, indicating the important influence of P on the biogeochemical cycle in the alpine ecosystem. Tang et al. (2016) also reported that the soil’s available P was strongly correlated with all N cycling genes except *amoA*, *AOB*, and *nirS*, suggesting that P plays a major role in regulating soil N cycling microbe. P limitation may occur in the growth of microorganisms because P-rich RNA needs P to synthesize proteins (He and Dijkstra, 2015). We speculate that P additions alleviate the P limitations of soil microorganisms and provide a more suitable environment for them. However, P addition showed a non-significant influence on microbial functional genes abundance when N was added, implying that N addition overwhelmed the positive effects of P on the abundance of microbial functional genes.

### Relationship Between Microbial Functional Genes and Environmental Variables

The structure and the function of the soil microbial community can be shaped by soil properties and plant traits (Li et al., 2017; Wang et al., 2017). In this study, the Mantel test showed that NO_3_^–^ and P_shoot_ significantly correlated with the whole community structure of C, N, and P cycling genes. In general, N availability can regulate the abundance, composition, and functional diversity of soil microbial microorganisms (Frey et al., 2004), because it directly restricts microbial growth (Treseder, 2010). Cong et al. (2015) demonstrated the key role of soil available N in controlling soil microbial functional gene structure and metabolic potential in a tropical rainforest. Combined with the results of VPA, P_shoot_ appeared to be the main factor altering the microbial functional communities in this study, because the effects of NO_3_^–^ alone were weak (Figure 8). In this study, P_shoot_ positively correlated with many C, N, and P cycling genes (Supplementary Figures 2–4). Based on this, we speculate that plant traits have indirect effects on soil microbial function genes. Cong et al. (2015) also reported that plant communities are major players in shaping microbial community structure. Moreover, a previous study in a semi-arid grassland ecosystem demonstrated the indirect effects of plant biomass on soil microbial functional genes (Li et al., 2017), supporting the strong links between plant traits and soil microbial community. We proposed that the increase in P_shoot_ induced by P addition would indirectly promote microbial functional genes through increasing rhizosphere secretion and C inputs to the soil, because plant aboveground traits, as well as symbiosis and exudation, can alter soil abiotic factors that regulate the abundance, composition, and activity of soil microbial communities (Abalos et al., 2019). Not only plant traits are important for the structure and function of soil microbial populations but the plant-microorganism interaction also can influence the composition of the microbial community.

## Conclusion

This study examined the responses of the C, N, and P biogeochemical cycle to N and P additions at the functional gene level in the alpine meadow. The results showed that N and P additions had inconsistent effects on the abundances of microbial functional genes. N addition had no significant effects on the abundances of C, N, and P cycling genes, while P addition significantly increased most of them, suggesting that soil microbial communities were more sensitive to P availability than to N availability in this alpine ecosystem. Combined N and P additions obviously decreased the abundances of microbial functional genes compared to P alone addition, which may be due to the overwhelming effects of N on P. The results confirmed the key role of P in C, N, and P cycling processes. P addition may increase microbial functional genes by enhancing P_shoot_ because P_shoot_ explained a large part of the variance of microbial functional genes. In future studies, the important role of plant traits in regulating the biogeochemical cycle should be addressed under N and P additions in the alpine grassland ecosystem.

## Data Availability Statement

The original contributions presented in the study are included in the article/Supplementary Material, further inquiries can be directed to the corresponding author.

## Author Contributions

JX: conceptualization, investigation, writing-original draft, and visualization. SD: supervision, resources, and funding acquisition. HS and SL: project administration and investigation. SL and WL: project administration, and writing-review. YZ and ZM: resources and methodology. KW and HL: writing-review and editing. All authors contributed to the article and approved the submitted version.

## Conflict of Interest

The authors declare that the research was conducted in the absence of any commercial or financial relationships that could be construed as a potential conflict of interest.

## Publisher’s Note

All claims expressed in this article are solely those of the authors and do not necessarily represent those of their affiliated organizations, or those of the publisher, the editors and the reviewers. Any product that may be evaluated in this article, or claim that may be made by its manufacturer, is not guaranteed or endorsed by the publisher.
