# Supplementary material for: N Addition Overwhelmed the Effects of P Addition on the Soil C, N, and P Cycling Genes in Alpine Meadow of the Qinghai-Tibetan Plateau
Source: Front Plant Sci. 2022 Apr 26;13:860590. doi: 10.3389/fpls.2022.860590 (PMC9087854; doi:10.3389/fpls.2022.860590)
Supplement: Supplementary file 1 [file Data_Sheet_2.docx]

**Supplementary materials**

**Table S1**. List of primers used in this study

| Gene Name | Forward Primer | Reverse Primer |
| --- | --- | --- |
| *rbcL* | AAGGACGACGAGAACATC | TGCAGGATCATGTCGTT |
| *aclB* | TGGACMATGGTDGCYGGKGGT | ATAGTTKGGSCCACCTCTTC |
| *korA* | GCCGGCTACCCCATCACCCC | ATGATGGGATGGTCCCCATG |
| *acsA* | GATACCTGGTGGCAGACCGA | TGATCACGTCGTCGACCCGG |
| *acsE* | TCATCGGCGAACGCATCAAC | AGRCCGGCTTCSATGGC |
| *acsB* | CTYTGYCAGTCMTTYGCBCC | CCCATAAABCCYGGDGTYTG |
| *accA* | GAAGGCTAYCGCAARGC | CCTTCMGGSGARATMAC |
| *pccA* | GTGMTGATCAAGGCCWC | CGSGTGTTCATYTCSAGGAA |
| *smtA* | TTTCTGGCCGGBTAYGCDGC | CGGTACGGIICCGGTYTGVCC |
| *mct* | TGGGCGCSGASGTSATMCG | TTCACSGTRTARTCSAYSGC |
| *frdA* | MTGCTGCACACSCTGTW | CCGGTSGGGTGRWACTG |
| *gam* | CGSAACTGGGAYTACCGS | TCCCACAGCCSKCGTC |
| *abfA* | CGSTAYCCSGCCGGCAAYTT | TGCCASGGNCCGTCCATYTC |
| *xylA* | TGGGGBGGTCGYGAAGG | ACTTTGGCRTCRAAGTT |
| *cxg* | YSTACGGSATGCACTGGMT | TANCGCAGRTAGTCVCCCAT |
| *exoPG* | ANCATTGGTGGCCSTGGAA | TTRAYGGCRATRCARTCRTC |
| *mnp* | MACRCCSTTCGACTCSACC | ACGTCSGAGCAGTCRAYGA |
| *Isop* | GTCATYTACTTYGGNCC | CGNGCSACATCNGCCCA |
| *apu* | ACVTGGATAGGYGAGCCYCA | CCRTCSGGGAAGTAGTTKCC |
| *amyA* | YGGTTTTCGTCTTGACGCSG | MGGCTGMGTRTCATGRTTK |
| *manA* | ATGCGCGGBGTCAACCA | TCGTTGSCGATGTTGABGA |
| *cdh* | ATWRYCTWCCGMRTHGCCMT | GTKAGSGGRTTBYKGRYCAT |
| *chiA* | TSAAGAARTACGCSGACAACG | ASGTCATCAGRCCCTTSAG |
| *glx* | AACCARTCGATCATCTACGA | RTGSACGAGCTCDGGCATGG |
| *lig* | CCGCACACACTGTTGCTGC | CGAAGGATTGCCACTCGCA |
| *pox* | ACYAGTATCCATTGGCACGGT | AGATGVGARTGATACCARAA |
| *mcrA* | GGTGGTGTMGGDTTCACMCARTA | CGTTCATBGCGTAGTTVGGRTAGT |
| *mmox* | ATGGAGGCGGTCAAGGACGA | CGCTTCATGCCCTTCCACAG |
| *pmoA* | GGNGACTGGGACTTCTGG | GAASGCNGAGAAGAASGC |
| *mxa* | GCGGCACCAACTGGGGCTGGT | GGGCAGCATGAAGGGCTCCC |
| *gdh* | GCCATCGGYCCWTACAAGGG | ATGTCRCCNGCCGGAACGTC |
| *ureC* | AAGMTSCACGAGGACTGGGG | AGRTGGTGGCASACCATSAGCAT |
| *nifH* | AAAGGYGGWATCGGYAARTCCACCAC | TGSGCYTTGTCYTCRCGGATBGGCAT |
| *hzsB* | ARGGHTGGGGHAGYTGGAAG | GTYCCHACRTCATGVGTCTG |
| *amoA1* | STAATGGTCTGGCTTAGACG | GCGGCCATCCATCTGTATGT |
| *amoB* | TGGTACGACATGATATGG | ACGCGGCAGGAACATCGG |
| *nxrA* | CAGACCGACGTGTGCGAAAG | TCCACAAGGAACGGAAGGTC |
| *nirS1* | GTSAACGTSAAGGARACSGG | GASTTCGGRTGSGTCTTGA |
| *nirS2* | ATCGTCAACGTCAARGARACVGG | TTCGGGTGCGTCTTSABGAASAG |
| *nirS3* | TGGAGGAACGCCGGNCARGTNTGG | GATGATGTCCACGGCNACRTANGG |
| *nirK1* | GGMATGGTKCCSTGGCA | GCCTCGATCAGRTTRTGGTT |
| *nirK2* | ATGGCGCCATCATGGTNYTNCC | TCHAAHHCCTCHATNARRTTRTG |
| *nirK3* | TGCACATCGCCAACGGNATGTWYGG | GGCGCGGAAGATGSHRTGRTCNAC |
| *nosZ1* | CGYTGTTCMTCGACAGCCAG | CGSACCTTSTTGCCSTYGCG |
| *nosZ2* | CGCRACGGCAASAAGGTSMSSGT | CAKRTGCAKSGCRTGGCAGAA |
| *ppx* | TGCATCTGGCGGACGGCCT | AGATCCHCCHCCAATATCA |
| *phoD* | CAGTGGGACGACCACGAGGT | GAGGCCGATCGGCATGTCG |
| *phoX* | GARGAGAACWTCCACGGYTA | GATCTCGATGATRTGRCCRAAG |
| *bpp* | GACGCAGCCGAYGAYCCNGCNITNTGG | CAGGSCGCANRTCIACRTTRTT |
| *pqqC* | AACCGCTTCTACTACCAG | GCGAACAGCTCGGTCAG |
| *gmGDH* | ATCGCGTTCGGGCCGGACG | ATSAGRTTSAGCTCGTCCCA |
| *emGDH* | TCTTCTATGTGCCGGCCAA | CTTCCACAGTTCCTTGCC |
| *phnK* | CATCGTCGGCGAATCCGG | TGCTGCATGCCGCCGGAAAA |
| 16SrRNA | GGGTTGCGCTCGTTGC | ATGGYTGTCGTCAGCTCGTG |

**Table S2**. Results of Two-way ANOVA on the effects of N addition (N), P addition (P), and their interactions on soil C, N and P cycling genes.

| Gene | N | P | N*P | Gene | N | P | N*P |
| --- | --- | --- | --- | --- | --- | --- | --- |
| korA | 0.91 | 9.74** | 7.42* | nifH | 2.36 | 10.6** | 7.21* |
| acsE | 0.13 | 12.86** | 6.77* | gdh | 1.09 | 10.44** | 4.99* |
| accA | 1.08 | 3.19 | 1.88 | UreC | 0.33 | 2.95 | 14.6** |
| aclB | 1.93 | 6.97* | 4.71* | amoA1 | 0.92 | 12.06** | 3.08 |
| acsA | 1.11 | 7.02* | 6.57* | amoB | 1.50 | 12.8** | 4.59* |
| frdA | 0.28 | 3.95 | 4.84* | hzsB | 0.15 | 12.18** | 14.21** |
| mct | 1.89 | 7.57* | 4.39* | nxrA | 8.25 | 1.69 | 1.32 |
| pccA | 1.38 | 5.44* | 4.00 | nirK1 | 0.72 | 3.48 | 3.81 |
| rbcL | 0.00 | 3.12 | 3.74 | nirK2 | 0.73 | 1.55 | 3.43 |
| smtA | 1.92 | 6.7* | 5.19* | nirK3 | 3.29 | 8.44** | 4.34* |
| amyA | 1.42 | 4.24* | 1.88 | nirS1 | 1.48 | 14.07** | 4.48* |
| apu | 0.73 | 9.8** | 3.39 | nirS2 | 0.67 | 8.68** | 6.44* |
| abfA | 0.04 | 2.92 | 7.55* | nirS3 | 1.22 | 12.22** | 4.33* |
| CDH | 0.12 | 11.07** | 7.49* | nosZ1 | 0.60 | 8.62** | 9.5** |
| chiA | 0.52 | 8.96** | 4.10 | nosZ2 | 3.39 | 9.96** | 4.32* |
| exg | 0.50 | 5.69* | 6.88* | napA | 2.00 | 7.42* | 5.02* |
| exoPG | 1.14 | 15.85*** | 3.85 | phnK | 0.73 | 10.29** | 11.92** |
| gam | 1.08 | 12.35** | 6.16* | pqqC | 0.17 | 7.31* | 4.49* |
| glx | 2.56 | 7.53* | 14.73** | bpp | 1.26 | 7.84** | 2.18 |
| IsoP | 2.24 | 9.97** | 6.06* | phoD | 1.29 | 7.72** | 8.88** |
| lig | 0.15 | 9.45** | 4.5* | phoX | 2.33 | 10.22** | 6.84* |
| mnp | 1.08 | 9.7** | 9.75** | ppx | 0.90 | 10.03** | 9.47** |
| manA | 1.70 | 9.44** | 2.78 | gmGDH | 0.50 | 2.71 | 4.77* |
| xylA | 2.46 | 8.6** | 5.45* | emGDH | 3.01 | 13.11** | 4.69* |

Note: * p < 0.05; ** p < 0.01; *** p < 0.001.

**Table S3**. Non-parametric multivariate dissimilarity tests of functional gene profiles between different treatments.

|  | MRPP | | Adonis | | ANOSIM | |
| --- | --- | --- | --- | --- | --- | --- |
|  | δ | p | F | p | R | p |
| Among all treatments | 0.473 | **0.001** | 0.834 | **0.001** | 0.596 | **0.001** |
| CK vs. N | -0.019 | 0.607 | 0.024 | 0.607 | -0.038 | 0.581 |
| CK vs. NP | 0.016 | 0.215 | 0.098 | 0.194 | 0.027 | 0.238 |
| CK vs. P | 0.248 | **0.004** | 0.366 | **0.009** | 0.475 | **0.002** |
| NP vs. N | 0.330 | **0.002** | 0.544 | **0.001** | 0.545 | **0.002** |
| NP vs. P | 0.449 | **0.001** | 0.696 | **0.001** | 0.848 | **0.001** |

Note: MRPP, multi-response permutation procedures; Adonis, permutational multivariate analysis of variance using distance matrices; ANOSIM, analysis of similarity. Results presented are based on distance matrices calculated with Bray-Curtis index. P values <0.05 are in bold.


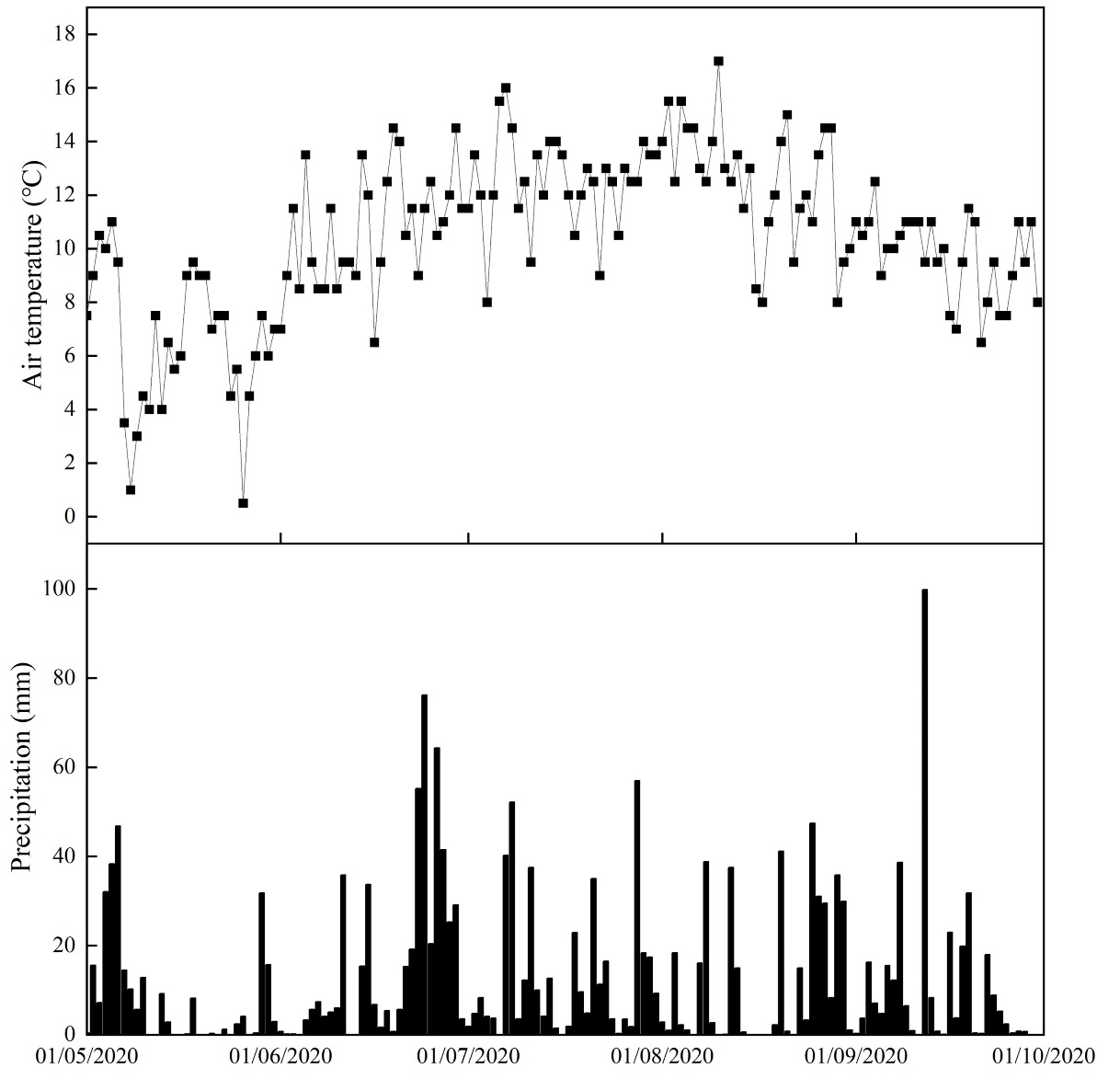


**Fig S1.** Mean daily air temperature and precipitation during the growth seasons.

**
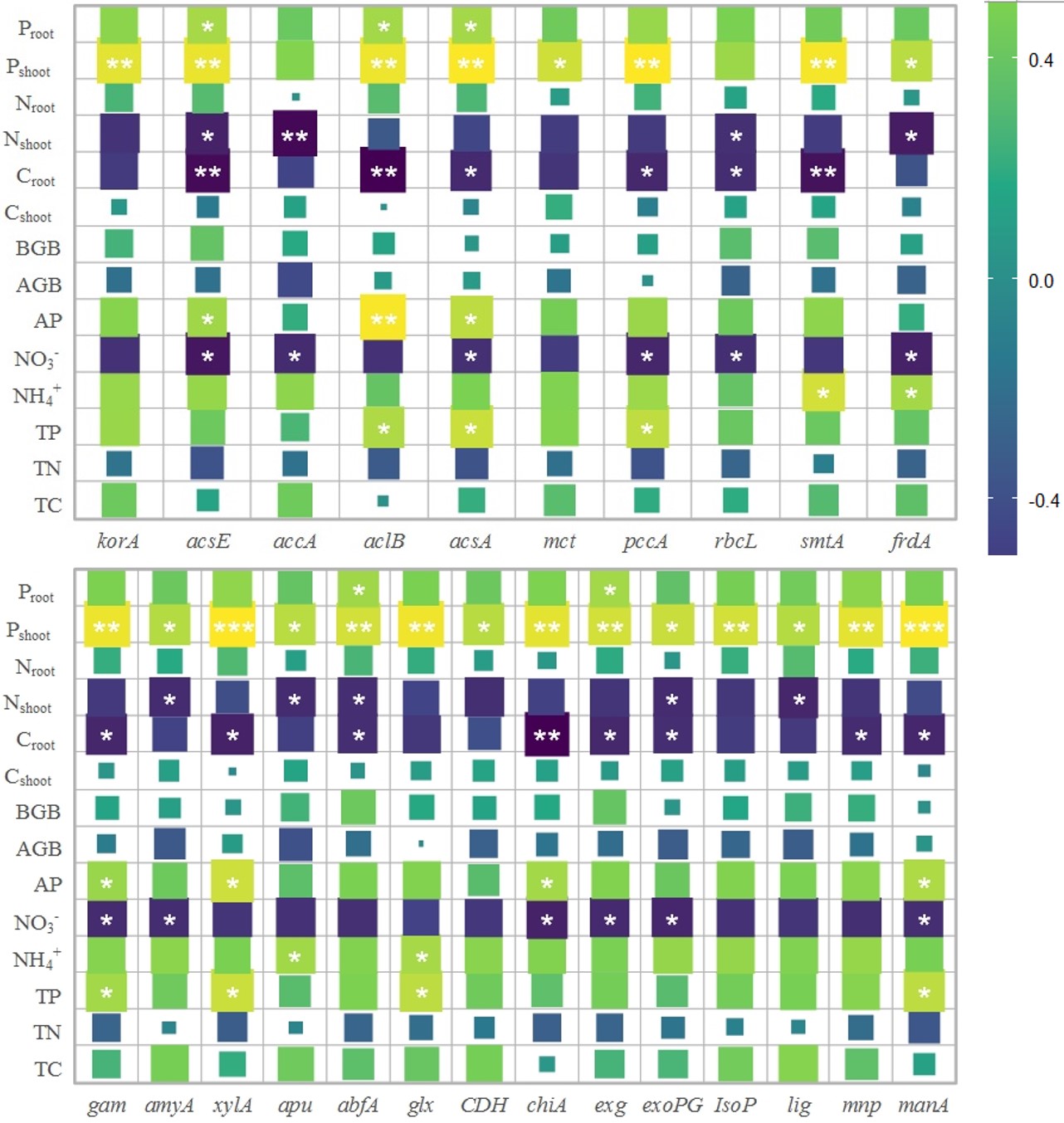
**

**Fig S2.** Pearson correlation analysis between C cycling genes and environmental variables, including soil TC, TN, TP, NH_4_^+^, NO_3_^-^, AP, plant AGB, BGB, C_shoot_, C_root_, N_shoot_, N_root_, P_root_, and P_root_.

**
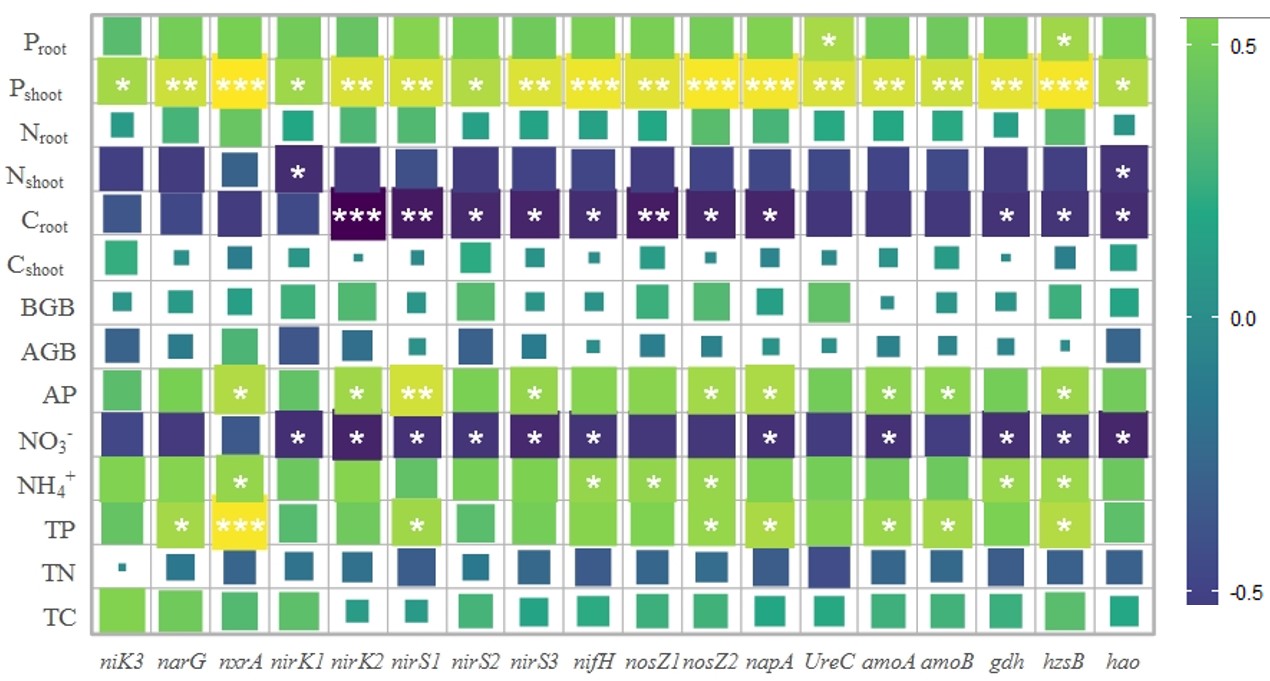
**

**Fig S3.** Pearson correlation analysis between N cycling genes and environmental variables, including soil TC, TN, TP, NH_4_^+^, NO_3_^-^, AP, plant AGB, BGB, C_shoot_, C_root_, N_shoot_, N_root_, P_root_, and P_root_.

**
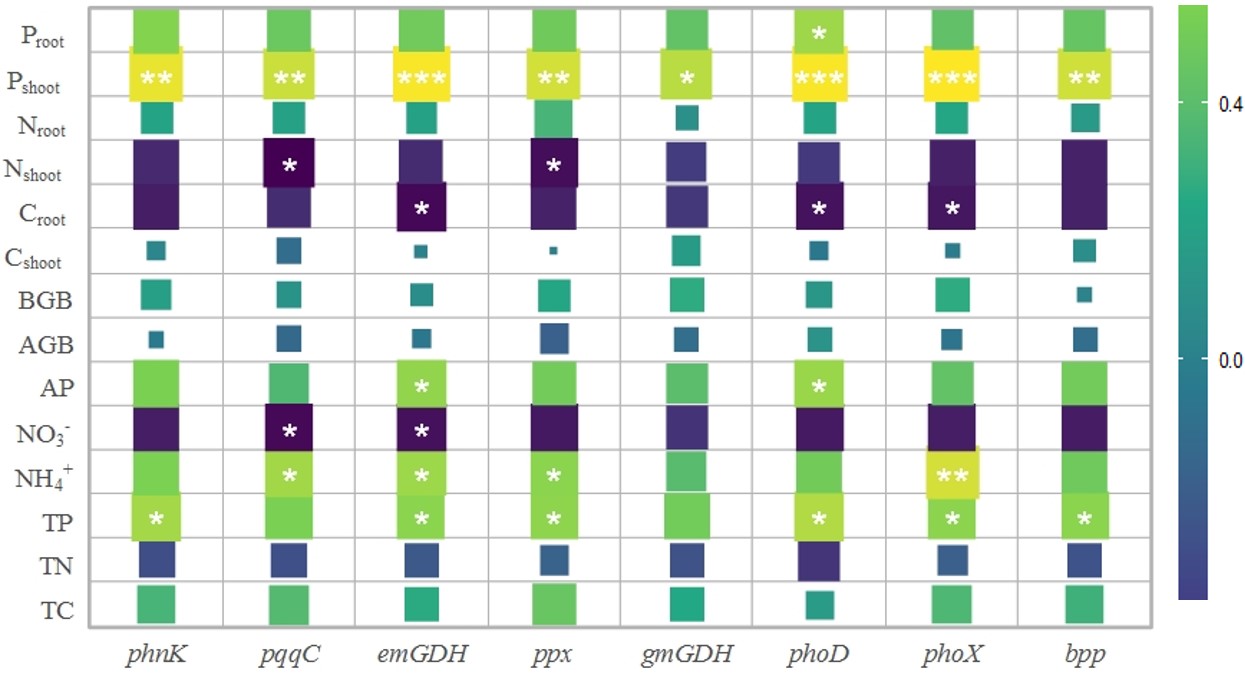
**

**Fig S4.** Pearson correlation analysis between P cycling genes and environmental variables, including soil TC, TN, TP, NH_4_^+^, NO_3_^-^, AP, plant AGB, BGB, C_shoot_, C_root_, N_shoot_, N_root_, P_root_, and P_root_.
